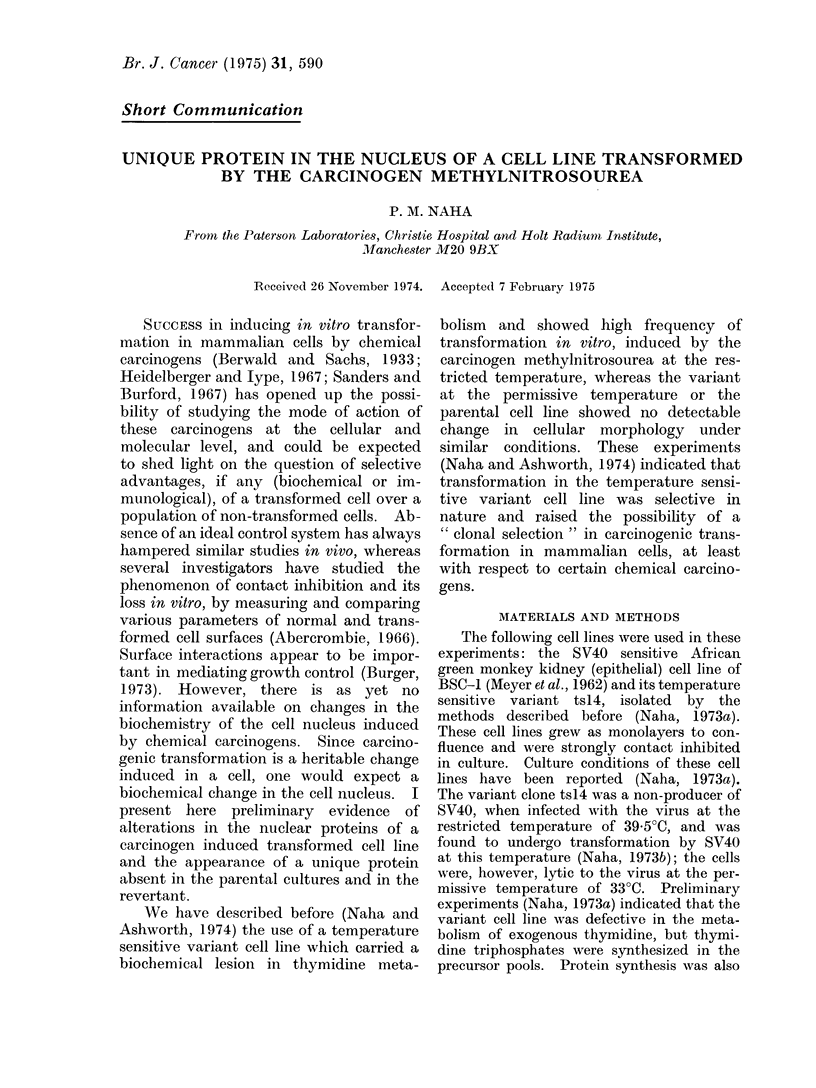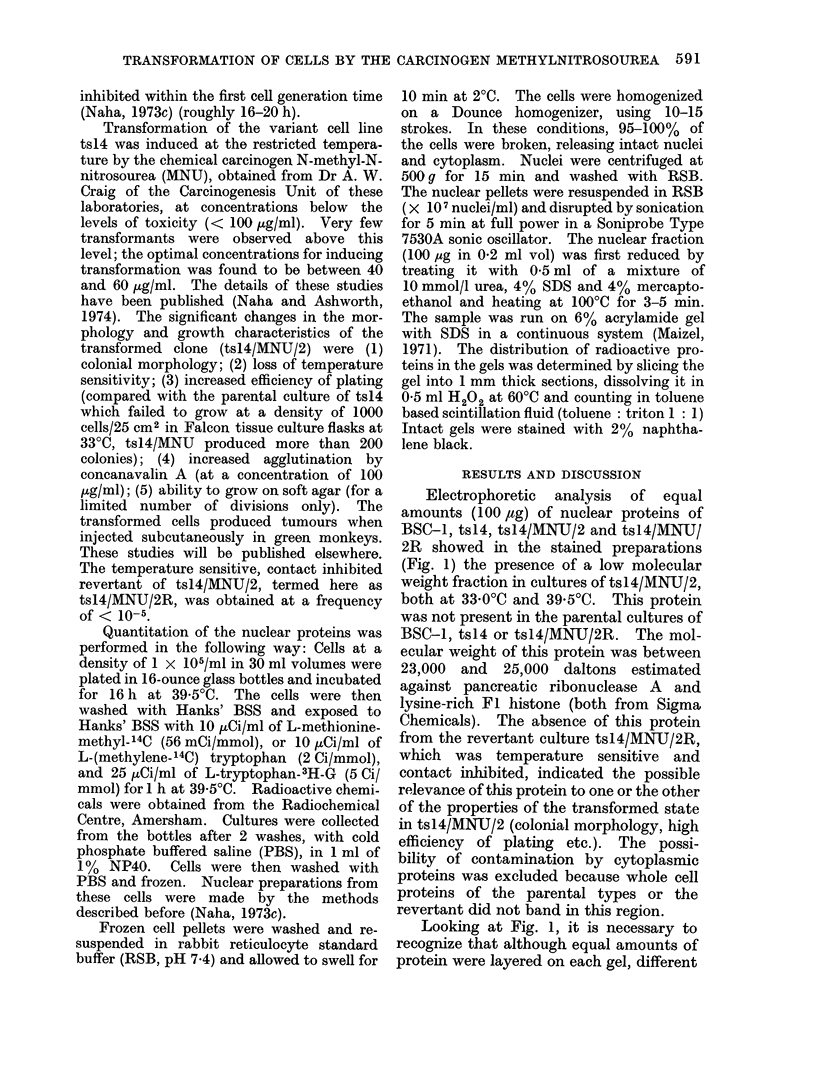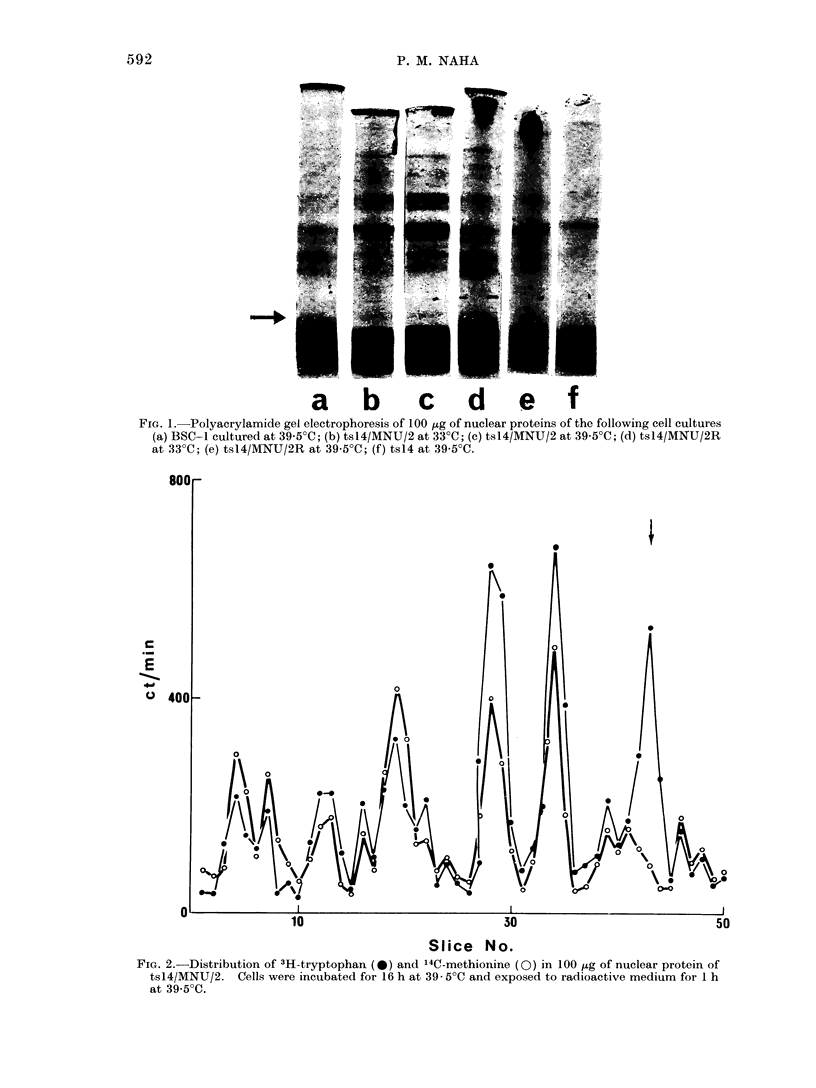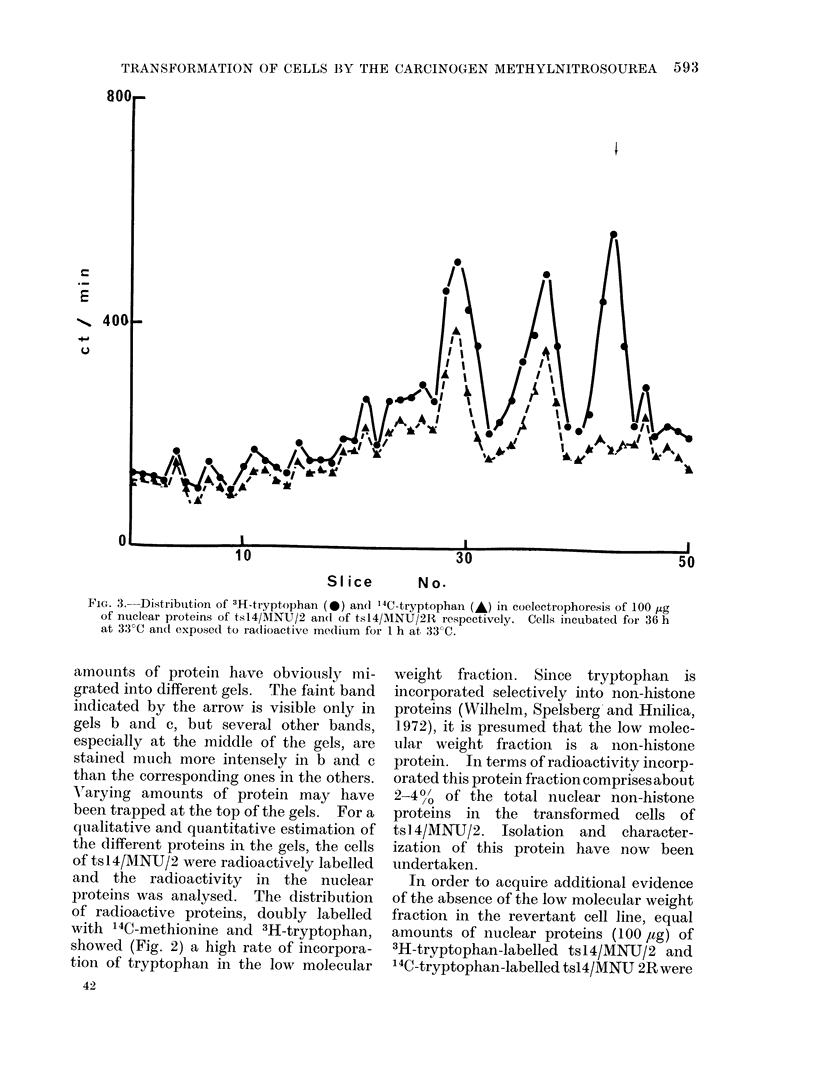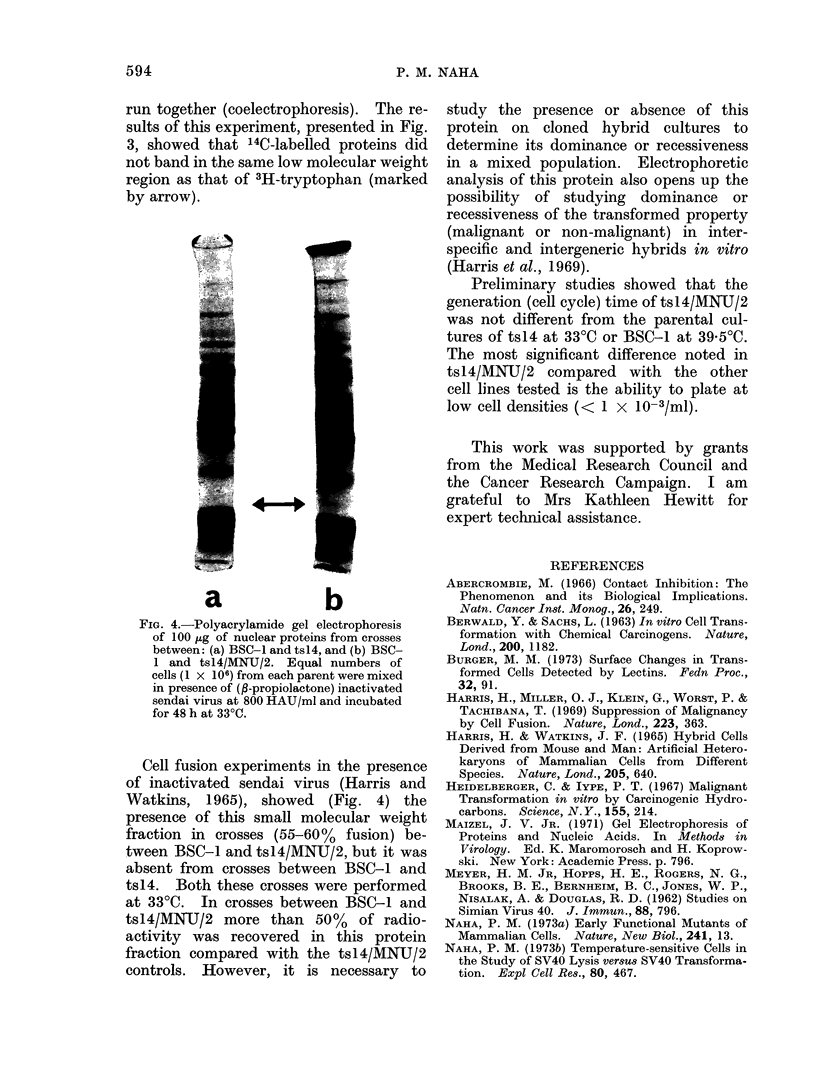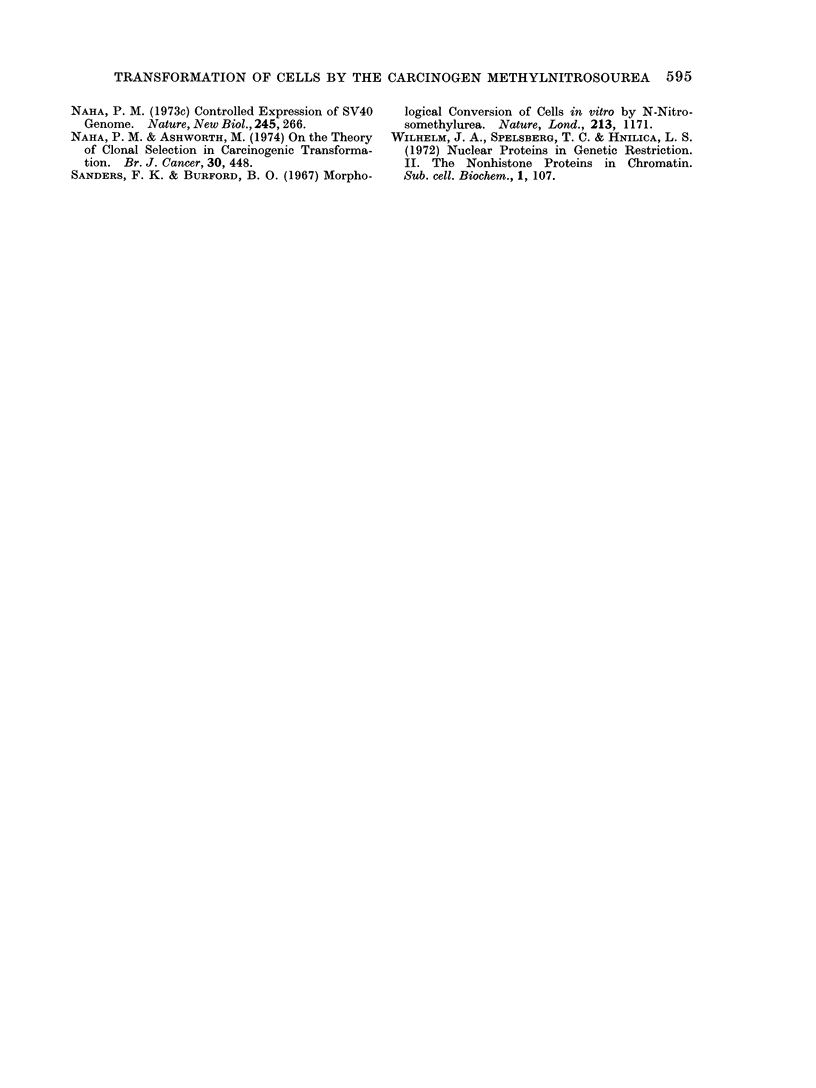# Unique protein in the nucleus of a cell line transformed by the carcinogen methylnitrosourea.

**DOI:** 10.1038/bjc.1975.102

**Published:** 1975-05

**Authors:** P. M. Naha

## Abstract

**Images:**


*Br. J. Cancer* (1975) **31**, 590

### *Short Communication*

### UNIQUE PROTEIN IN THE NUCLEUS OF A CELL LINE TRANSFORMED BY THE CARCINOGEN METHYLNITROSOUREA

P. M. NAHA

*From the Paterson Laboratories, Christie Hospital and Holt Radium Institute, Manchester M*20 9*BX*

Received 26 November 1974. Accepted 7 February 1975

SUCCESS in inducing *in vitro* transformation in mammalian cells by chemical carcinogens (Berwald and Sachs, 1933; Heidelberger and Iype, 1967; Sanders and Burford, 1967) has opened up the possibility of studying the mode of action of these carcinogens at the cellular and molecular level, and could be expected to shed light on the question of selective advantages, if any (biochemical or immunological), of a transformed cell over a population of non-transformed cells. Absence of an ideal control system has always hampered similar studies *in vivo*, whereas several investigators have studied the phenomenon of contact inhibition and its loss *in vitro*, by measuring and comparing various parameters of normal and transformed cell surfaces (Abercrombie, 1966). Surface interactions appear to be important in mediating growth control (Burger, 1973). However, there is as yet no information available on changes in the biochemistry of the cell nucleus induced by chemical carcinogens. Since carcinogenic transformation is a heritable change induced in a cell, one would expect a biochemical change in the cell nucleus. I present here preliminary evidence of alterations in the nuclear proteins of a carcinogen induced transformed cell line and the appearance of a unique protein absent in the parental cultures and in the revertant.

We have described before (Naha and Ashworth, 1974) the use of a temperature sensitive variant cell line which carried a biochemical lesion in thymidine metabolism and showed high frequency of transformation *in vitro*, induced by the carcinogen methylnitrosourea at the restricted temperature, whereas the variant at the permissive temperature or the parental cell line showed no detectable change in cellular morphology under similar conditions. These experiments (Naha and Ashworth, 1974) indicated that transformation in the temperature sensitive variant cell line was selective in nature and raised the possibility of a " clonal selection " in carcinogenic transformation in mammalian cells, at least with respect to certain chemical carcinogens.

### MATERIALS AND METHODS

The following cell lines were used in these experiments: the SV40 sensitive African green monkey kidney (epithelial) cell line of BSC-1 (Meyer *et al.*, 1962) and its temperature sensitive variant ts14, isolated by the methods described before (Naha, 1973*a*). These cell lines grew as monolayers to confluence and were strongly contact inhibited in culture. Culture conditions of these cell lines have been reported (Naha, 1973*a*). The variant clone ts14 was a non-producer of SV40, when infected with the virus at the restricted temperature of 39·5°C, and was found to undergo transformation by SV40 at this temperature (Naha, 1973*b*); the cells were, however, lytic to the virus at the permissive temperature of 33°C. Preliminary experiments (Naha, 1973*a*) indicated that the variant cell line was defective in the metabolism of exogenous thymidine, but thymidine triphosphates were synthesized in the precursor pools. Protein synthesis was also inhibited within the first cell generation time (Naha, 1973*c*) (roughly 16–20 h).

Transformation of the variant cell line ts14 was induced at the restricted temperature by the chemical carcinogen N-methyl-N-nitrosourea (MNU), obtained from Dr A. W. Craig of the Carcinogenesis Unit of these laboratories, at concentrations below the levels of toxicity ($< 100\ \mu g/ml$). Very few transformants were observed above this level; the optimal concentrations for inducing transformation was found to be between 40 and 60 $\mu$g/ml. The details of these studies have been published (Naha and Ashworth, 1974). The significant changes in the morphology and growth characteristics of the transformed clone (ts14/MNU/2) were (1) colonial morphology; (2) loss of temperature sensitivity; (3) increased efficiency of plating (compared with the parental culture of ts14 which failed to grow at a density of 1000 cells/25 $cm^2$ in Falcon tissue culture flasks at 33°C, ts14/MNU produced more than 200 colonies); (4) increased agglutination by concanavalin A (at a concentration of 100 $\mu$g/ml); (5) ability to grow on soft agar (for a limited number of divisions only). The transformed cells produced tumours when injected subcutaneously in green monkeys. These studies will be published elsewhere. The temperature sensitive, contact inhibited revertant of ts14/MNU/2, termed here as ts14/MNU/2R, was obtained at a frequency of $< 10^{-5}$.

Quantitation of the nuclear proteins was performed in the following way: Cells at a density of $1 \times 10^5$/ml in 30 ml volumes were plated in 16-ounce glass bottles and incubated for 16 h at 39·5°C. The cells were then washed with Hanks' BSS and exposed to Hanks' BSS with 10 $\mu$Ci/ml of L-methionine-methyl-$^{14}$C (56 mCi/mmol), or 10 $\mu$Ci/ml of L-(methylene-$^{14}$C) tryptophan (2 Ci/mmol), and 25 $\mu$Ci/ml of L-tryptophan-$^3$H-G (5 Ci/mmol) for 1 h at 39·5°C. Radioactive chemicals were obtained from the Radiochemical Centre, Amersham. Cultures were collected from the bottles after 2 washes, with cold phosphate buffered saline (PBS), in 1 ml of 1% NP40. Cells were then washed with PBS and frozen. Nuclear preparations from these cells were made by the methods described before (Naha, 1973*c*).

Frozen cell pellets were washed and resuspended in rabbit reticulocyte standard buffer (RSB, pH 7·4) and allowed to swell for 10 min at 2°C. The cells were homogenized on a Dounce homogenizer, using 10–15 strokes. In these conditions, 95–100% of the cells were broken, releasing intact nuclei and cytoplasm. Nuclei were centrifuged at 500 *g* for 15 min and washed with RSB. The nuclear pellets were resuspended in RSB ($\times 10^7$ nuclei/ml) and disrupted by sonication for 5 min at full power in a Soniprobe Type 7530A sonic oscillator. The nuclear fraction (100 $\mu$g in 0·2 ml vol) was first reduced by treating it with 0·5 ml of a mixture of 10 mmol/l urea, 4% SDS and 4% mercaptoethanol and heating at 100°C for 3–5 min. The sample was run on 6% acrylamide gel with SDS in a continuous system (Maizel, 1971). The distribution of radioactive proteins in the gels was determined by slicing the gel into 1 mm thick sections, dissolving it in 0·5 ml $H_2O_2$ at 60°C and counting in toluene based scintillation fluid (toluene : triton 1 : 1) Intact gels were stained with 2% naphthalene black.

### RESULTS AND DISCUSSION

Electrophoretic analysis of equal amounts (100 $\mu$g) of nuclear proteins of BSC–1, ts14, ts14/MNU/2 and ts14/MNU/2R showed in the stained preparations (Fig. 1) the presence of a low molecular weight fraction in cultures of ts14/MNU/2, both at 33·0°C and 39·5°C. This protein was not present in the parental cultures of BSC–1, ts14 or ts14/MNU/2R. The molecular weight of this protein was between 23,000 and 25,000 daltons estimated against pancreatic ribonuclease A and lysine-rich F1 histone (both from Sigma Chemicals). The absence of this protein from the revertant culture ts14/MNU/2R, which was temperature sensitive and contact inhibited, indicated the possible relevance of this protein to one or the other of the properties of the transformed state in ts14/MNU/2 (colonial morphology, high efficiency of plating etc.). The possibility of contamination by cytoplasmic proteins was excluded because whole cell proteins of the parental types or the revertant did not band in this region.

Looking at Fig. 1, it is necessary to recognize that although equal amounts of protein were layered on each gel, different

FIG. 1.—Polyacrylamide gel electrophoresis of 100 μg of nuclear proteins of the following cell cultures (a) BSC-1 cultured at 39·5°C; (b) ts14/MNU/2 at 33°C; (c) ts14/MNU/2 at 39·5°C; (d) ts14/MNU/2R at 33°C; (e) ts14/MNU/2R at 39·5°C; (f) ts14 at 39·5°C.

FIG. 2.—Distribution of $^{3}$H-tryptophan (●) and $^{14}$C-methionine (○) in 100 μg of nuclear protein of ts14/MNU/2. Cells were incubated for 16 h at 39·5°C and exposed to radioactive medium for 1 h at 39·5°C.

FIG. 3.—Distribution of $^{3}$H-tryptophan (●) and $^{14}$C-tryptophan (▲) in coelectrophoresis of 100 μg of nuclear proteins of ts14/MNU/2 and of ts14/MNU/2R respectively. Cells incubated for 36 h at 33°C and exposed to radioactive medium for 1 h at 33°C.

amounts of protein have obviously migrated into different gels. The faint band indicated by the arrow is visible only in gels b and c, but several other bands, especially at the middle of the gels, are stained much more intensely in b and c than the corresponding ones in the others. Varying amounts of protein may have been trapped at the top of the gels. For a qualitative and quantitative estimation of the different proteins in the gels, the cells of ts14/MNU/2 were radioactively labelled and the radioactivity in the nuclear proteins was analysed. The distribution of radioactive proteins, doubly labelled with $^{14}$C-methionine and $^{3}$H-tryptophan, showed (Fig. 2) a high rate of incorporation of tryptophan in the low molecular weight fraction. Since tryptophan is incorporated selectively into non-histone proteins (Wilhelm, Spelsberg and Hnilica, 1972), it is presumed that the low molecular weight fraction is a non-histone protein. In terms of radioactivity incorporated this protein fraction comprises about 2–4% of the total nuclear non-histone proteins in the transformed cells of ts14/MNU/2. Isolation and characterization of this protein have now been undertaken.

In order to acquire additional evidence of the absence of the low molecular weight fraction in the revertant cell line, equal amounts of nuclear proteins (100 μg) of $^{3}$H-tryptophan-labelled ts14/MNU/2 and $^{14}$C-tryptophan-labelled ts14/MNU 2R were run together (coelectrophoresis). The results of this experiment, presented in Fig. 3, showed that $^{14}$C-labelled proteins did not band in the same low molecular weight region as that of $^{3}$H-tryptophan (marked by arrow).

FIG. 4.—Polyacrylamide gel electrophoresis of 100 $\mu$g of nuclear proteins from crosses between: (a) BSC–1 and ts14, and (b) BSC–1 and ts14/MNU/2. Equal numbers of cells ($1 \times 10^6$) from each parent were mixed in presence of ($\beta$-propiolactone) inactivated sendai virus at 800 HAU/ml and incubated for 48 h at 33°C.

Cell fusion experiments in the presence of inactivated sendai virus (Harris and Watkins, 1965), showed (Fig. 4) the presence of this small molecular weight fraction in crosses (55–60% fusion) between BSC–1 and ts14/MNU/2, but it was absent from crosses between BSC–1 and ts14. Both these crosses were performed at 33°C. In crosses between BSC–1 and ts14/MNU/2 more than 50% of radioactivity was recovered in this protein fraction compared with the ts14/MNU/2 controls. However, it is necessary to study the presence or absence of this protein on cloned hybrid cultures to determine its dominance or recessiveness in a mixed population. Electrophoretic analysis of this protein also opens up the possibility of studying dominance or recessiveness of the transformed property (malignant or non-malignant) in interspecific and intergeneric hybrids *in vitro* (Harris *et al.*, 1969).

Preliminary studies showed that the generation (cell cycle) time of ts14/MNU/2 was not different from the parental cultures of ts14 at 33°C or BSC–1 at 39·5°C. The most significant difference noted in ts14/MNU/2 compared with the other cell lines tested is the ability to plate at low cell densities ($< 1 \times 10^{-3}$/ml).

This work was supported by grants from the Medical Research Council and the Cancer Research Campaign. I am grateful to Mrs Kathleen Hewitt for expert technical assistance.

### REFERENCES

ABERCROMBIE, M. (1966) Contact Inhibition: The Phenomenon and its Biological Implications. *Natn. Cancer Inst. Monog.*, **26**, 249.

BERWALD, Y. & SACHS, L. (1963) *In vitro* Cell Transformation with Chemical Carcinogens. *Nature, Lond.*, **200**, 1182.

BURGER, M. M. (1973) Surface Changes in Transformed Cells Detected by Lectins. *Fedn Proc.*, **32**, 91.

HARRIS, H., MILLER, O. J., KLEIN, G., WORST, P. & TACHIBANA, T. (1969) Suppression of Malignancy by Cell Fusion. *Nature, Lond.*, **223**, 363.

HARRIS, H. & WATKINS, J. F. (1965) Hybrid Cells Derived from Mouse and Man: Artificial Heterokaryons of Mammalian Cells from Different Species. *Nature, Lond.*, **205**, 640.

HEIDELBERGER, C. & IYPE, P. T. (1967) Malignant Transformation *in vitro* by Carcinogenic Hydrocarbons. *Science, N.Y.*, **155**, 214.

MAIZEL, J. V. JR. (1971) Gel Electrophoresis of Proteins and Nucleic Acids. In *Methods in Virology*. Ed. K. Maromorosch and H. Koprowski. New York: Academic Press. p. 796.

MEYER, H. M. JR, HOPPS, H. E., ROGERS, N. G., BROOKS, B. E., BERNHEIM, B. C., JONES, W. P., NISALAK, A. & DOUGLAS, R. D. (1962) Studies on Simian Virus 40. *J. Immun.*, **88**, 796.

NAHA, P. M. (1973*a*) Early Functional Mutants of Mammalian Cells. *Nature, New Biol.*, **241**, 13.

NAHA, P. M. (1973*b*) Temperature-sensitive Cells in the Study of SV40 Lysis *versus* SV40 Transformation. *Expl Cell Res.*, **80**, 467.

Naha, P. M. (1973c) Controlled Expression of SV40 Genome. *Nature, New Biol.*, **245**, 266.

Naha, P. M. & Ashworth, M. (1974) On the Theory of Clonal Selection in Carcinogenic Transformation. *Br. J. Cancer*, **30**, 448.

Sanders, F. K. & Burford, B. O. (1967) Morphological Conversion of Cells *in vitro* by N-Nitrosomethylurea. *Nature, Lond.*, **213**, 1171.

Wilhelm, J. A., Spelsberg, T. C. & Hnilica, L. S. (1972) Nuclear Proteins in Genetic Restriction. II. The Nonhistone Proteins in Chromatin. *Sub. cell. Biochem.*, **1**, 107.